# The prevalence and impact of eating disorder behaviours in Australian men

**DOI:** 10.1186/2050-2974-1-S1-O23

**Published:** 2013-11-14

**Authors:** Deborah Mitchison, Jonathan Mond, Shameran Slewa-Younan, Phillipa Hay

**Affiliations:** 1 School of Medicine, University of Western Sydney, Australia; 2 Centre for Applied Psychology, University of Canberra, Australia; 3 School of Medicine, University of Western Sydney, Australia; 4 Centre for Health Research, School of Medicine, University of Western Sydney, Australia

## Objective

To determine sex differences in the prevalence and associated impairment of eating disorder (ED) features over time.

## Method

Cross-sectional surveys of randomly selected adults were carried out in 1998 (n = 3010) and 2008 (n = 3034). Outcomes included self-reported health-related quality of life (HRQoL), objective and subjective binge eating, extreme dieting, purging, and overevaluation of weight or shape.

## Results

Men represented 23 - 41% of participants who reported ED features. Objective binge eating was associated with greater reductions in mental HRQoL in men compared to women (p < 0.05), whereas overevaluation of weight or shape was associated with greater reductions in HRQoL in women compared to men (p < 0.05). The prevalence of extreme dieting and purging increased at a faster rate in men compared to women (p = 0.03), whereas the rate of increase in objective binge eating was similar between the sexes (p > 0.05). Mental HRQoL impairment associated with binge eating had increased over time for men but not for women (p < 0.05).

## Conclusions

The gender gap in the prevalence and impact of ED behaviours may be closing. Implications include the need for more gender-neutral public health campaigns and interventions, and the active inclusion of male participants in ED research.

This abstract was presented in the **Disordered Eating – Characteristics & Treatment** stream of the 2013 ANZAED Conference.

